# A Scoping Review on the Therapeutic Potential of Resin From the Species *Larix decidua* Mill. [Pinaceae] to Treat Ulcerating Wounds

**DOI:** 10.3389/fphar.2022.895838

**Published:** 2022-06-03

**Authors:** João V. C. Batista, Annekathrin Uecker, Carla Holandino, Fabio Boylan, Jakob Maier, Jörg Huwyler, Stephan Baumgartner

**Affiliations:** ^1^ Hiscia Institute, Society for Cancer Research, Arlesheim, Switzerland; ^2^ Department of Pharmaceutical Sciences, Division of Pharmaceutical Technology, University of Basel, Basel, Switzerland; ^3^ Institute of Integrative Medicine, University of Witten/Herdecke, Witten, Germany; ^4^ Departamento de Fármacos e Medicamentos, Faculdade de Farmácia, Universidade Federal do Rio de Janeiro, Rio de Janeiro, Brazil; ^5^ School of Pharmacy and Pharmaceutical Sciences, Trinity Biomedical Sciences Institute, Trinity College Dublin, Dublin, Ireland; ^6^ Institute of Complementary and Integrative Medicine, University of Bern, Bern, Switzerland

**Keywords:** Larch resin, *Larix decidua* Mill. [Pinaceae], phytochemistry, phytotherapy, wound healing

## Abstract

Malignant ulcerating wounds or neoplastic lesions are a considerable burden for patients suffering from advanced cancer. These wounds have no effective treatment and are very difficult to manage. The present review summarizes evidence in support of a hypothesis put forward in anthroposophic medicine, which suggests a beneficial role of resin from the species *Larix decidua* Mill. [Pinaceae] for treating such wounds. A systematic search strategy was performed using the databases PubMed, EMBASE and SciFinder. The included publications described the chemical composition of this species, as well as *in vitro*, *in vivo*, and *ex vivo* experiments using plant extracts and isolated compounds. The results show that among the phytochemical classes, terpenoids were the major components of this species, especially in the resin. The summarized biological experiments revealed antimicrobial, antioxidant and anti-inflammatory effects, with promising potential for the extracts and isolated compounds. However, the molecular mechanisms and toxicological effects are as of yet not conclusively evaluated. From the data of our study, we can conclude that *L. decidua* might indeed have a promising potential for the treatment of malignant wounds, but definitive information that can prove its effectiveness is still lacking. We therefore suggest that future efforts should be dedicated to the evaluation of *L. decidua* resin's therapeutic use considering its antiseptic action and proposed wound healing properties.

## 1 Introduction

In advanced cancer patients, palliative care becomes the primary focus, in an attempt to alleviate the pain, treat the symptoms and improve the patient’s comfort (Vardhan et al., 2019). Among the most distressing discomforts that such patients have to endure, malignant fungating wounds account for a prevalence of 5%–14%. Malignant fungating wounds occur due to an aggressive proliferation and infiltration caused by a local tumor or a metastatic spread into the skin, blood and lymph vessels, resulting in tissue damage, hypoxia, necrosis, microbial proliferation and fungating ulceration of the wound. They are commonly present in the following body areas: breast (66%), head and neck (24%), followed by the groin, genital and back (3%), and various tissues (8%) (Tsichlakidou et al., 2019; Vardhan et al., 2019; Tilley et al., 2020). In addition, they are characterised by presenting a malodour, exudates, bleeding, pain, itching, irritation, infection, and necrosis (Adderley and Holt, 2014; Vardhan et al., 2019). The effects of such wounds, also known as ulcerating wounds, malignant wounds or neoplastic lesions, cause physiological and psychological distress to the patients by affecting not only their wellbeing but also their social life. With respect to social life, it is known that due to the repellent malodor and the presence of exudates, patients are ashamed and therefore try to avoid social contact. This self-isolation leads to additional suffering and depression. At the same time, the treatment of these wounds remains a challenge (Regan, 2007; Adderley and Holt, 2014; Vardhan et al., 2019). Currently, options are limited and include the systemic and/or topical application of analgesics, antibiotics, and coagulants (Regan, 2007; Adderley and Holt, 2014).

Ethnobotanical studies in the Balkan region described the use of *L. decidua* bark, needles and resin for internal and external use, for blood purification, renal, urinary, and gallbladder stones, in addition to wound healing, ulcers, and restlessness treatment (Saric-Kundalic et al., 2011; Jaric et al., 2018). The Committee for Veterinary Medicinal Products from the European Medicines Agency approved *L. decidua* resin for topical application in animals. The concentration of the resin varies from 10% to 20% for the treatment of skin wounds and promotion of wound healing (EMA, 1998). The German Drugbase lists it as an external application for rheumatic and neuralgic disorders, also for catarrhal illness in humans (Drugbase, 2021). A prospective, randomized and controlled multicenter trial using resin from *Picea abies* (L.) H.Karst. included 37 patients in the treatment of pressure ulcers and the healing activity was observed in 92% within the treated group (Sipponen et al., 2008). Sipponen et al. (2012) included 23 patients in their study and saw a healing rate of complicated chronic surgical wounds of 100%, within a period of 43 ± 24 days. In addition, Goels et al. (2022) compared the wound healing potential of *P. abies*, *Pinus nigra* J.F.Arnold and *L. decidua in vitro*. The reduction of cell-free area in a keratinocyte wound healing assay was significant for the balm from *L. decidua* (26%) when compared to the *P. abies* balm and resin (16.7% and 9.6%, respectively) and to the *P. nigra* resin (16.2%). It is therefore the aim of the present review article to explore whether *L. decidua*´s resin (European larch tree), which has been used for wound healing for some time, as proposed in the context of anthroposophic medicine (Krüger, 1969), might offer new therapeutic options and therefore deserves to be investigated in more detail. Gaps in the existing knowledge were identified and addressed with respect to a systematic evaluation of *in vitro* and *in vivo* studies to justify the uses of this species, the standardized evaluation of pharmacological effects, limitations of existing studies, and prospects for future research and potential clinical applications.

## 2 Materials and Methods

Literature search was performed using MEDLINE (PubMed), EMBASE and SciFinder databases. This scoping review aims at identifying the nature and extent of research evidence using systematic, transparent and replicable characteristics for data collection, analysis and interpretation and subsequently providing an overview or map of evidence on the topic (Grant and Booth, 2009; Munn et al., 2018). The phases implemented in this scoping review were: 1) collection of relevant literature; 2) selection of publications based on pre-defined criteria; 3) extraction of relevant data; 4) describing and synthesising the findings. There was no initial period or language restriction for the search. Literature covers a time period up to 26 March 2021. “*Larix decidua*” was used as a single search keyword. The inclusion criteria comprised articles reporting on investigations of any kind of extracts from *L. decidua* related to its chemical composition, *in vitro, in vivo*, *ex vivo*, and clinical research, and ethnopharmacology. The exclusion criteria comprised: irrelevant outcome (genetic analysis, environmental behaviour, and wood properties), irrelevant sample (wood for construction, wood as furniture, and wood properties), insufficient data (results were not described for this species even though it was declared in the methods).

## 3 Results

### 3.1 Identification of Studies

**FIGURE 1:**
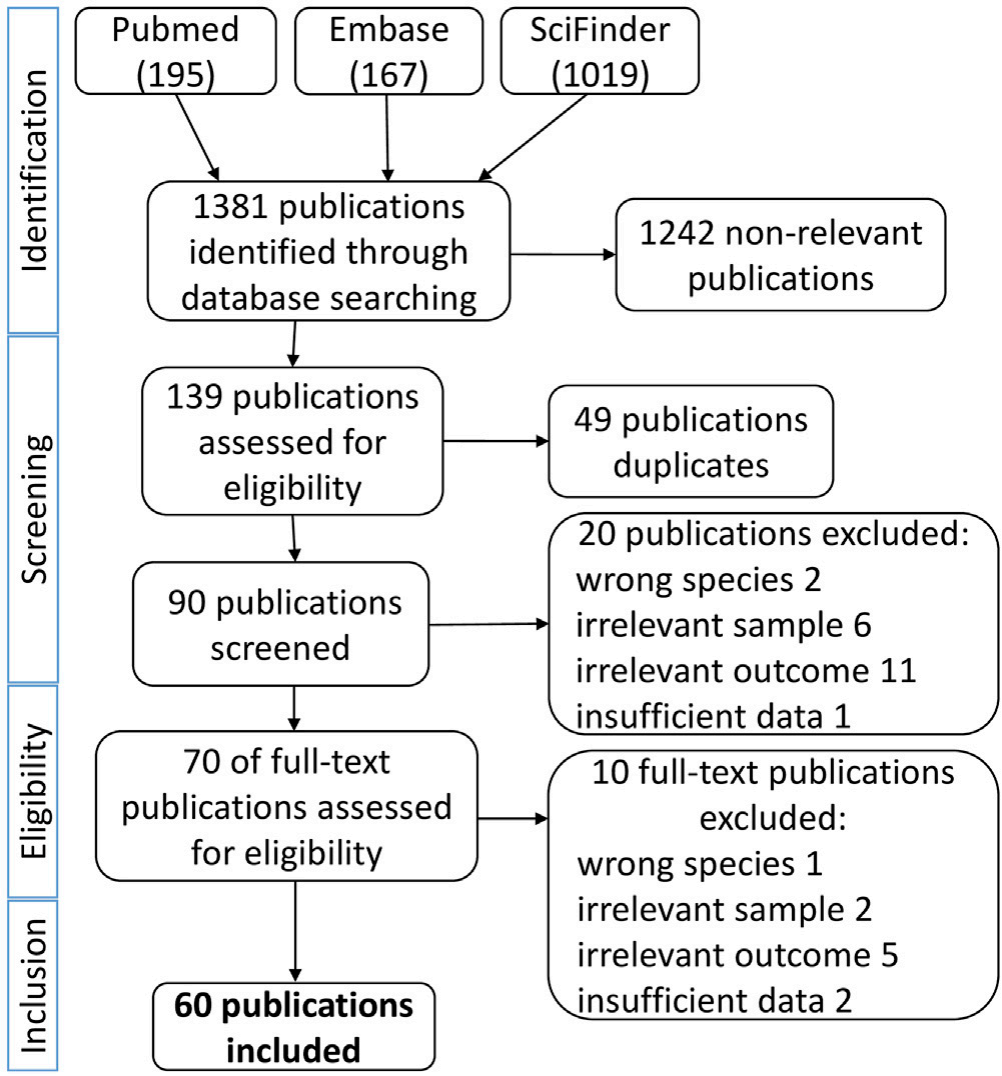
Methodological workflow from the databases to the final publications selected to be included in this review based on defined key words and exclusion criteria (see section *Materials and Methods*).

During the first phase of the literature search, *n* = 1,376 articles in English and *n* = 5 articles in German were identified. After a screening of the abstracts, 139 articles were assessed in more detail. After exclusion of 49 duplicates, *n* = 70 were considered to be eligible for a detailed full-text review after exclusion of studies with irrelevant outcome (*n* = 11), irrelevant sample size (*n* = 6), wrong species (*n* = 2), or insufficient data (*n* = 1). The 70 studies were categorized according to their field of research and/or outcome, such as *in vivo*, *ex vivo*, *in vitro* with biological approach, *in vitro* with chemical approach, *in vitro* with biological and chemical approaches, and chemistry. 10 publications were excluded after reading the full text due to technical shortcomings or lack of critical information (Figure 1). A total of 60 publications were finally identified as satisfying the inclusion criteria for full article evaluation. The whole selection process is represented in Figure 1.

**FIGURE 2:**
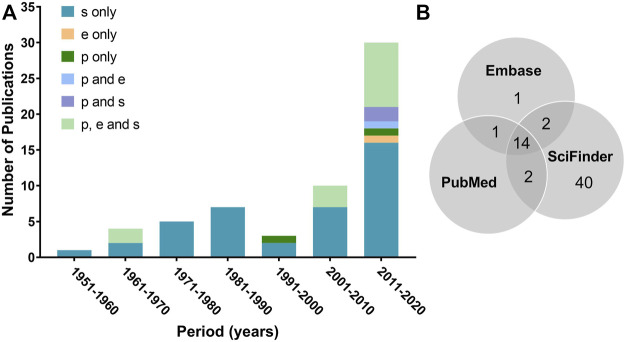
Total number of publications by year of publication **(A)** and by database **(B)**. Abbreviations: s, SciFinder; e, Embase; p, PubMed. Categories are color coded depending on findings in one defined database or entries found in several databases.

Included studies were published between 1952 and 2020, with 67% being published from 2001 onwards (Figure 2A). This demonstrates an increasing number of publications in later years and an increasing interest for the biological potential of *L. decidua* over time and in particular since 2016. Figure 2B illustrates the listing of articles in different databases. Most studies were categorized as “chemistry” (*n* = 42), followed by “*in vitro* with biological and chemical approaches” (*n* = 7), “*in vitro* with biological approaches” (*n* = 6), “*in vivo”* (*n* = 2), “*in vitro* with chemical approaches” (*n* = 2), and “*ex vivo”* (*n* = 1). These categories are in accordance to the higher number of publications found on SciFinder, which is a database for chemical literature.

### 3.2 Phytochemistry

The literature review showed that the majority of articles found for *L. decidua* relates to its chemical composition. Amongst all the publications in this review (*n* = 60), forty-two dealt with the chemical compounds found in different parts of the tree. Nine extra publications included the chemical analysis besides other *in vitro* pharmacological investigations. The first study is dated from 1952 and is the oldest publication included in the review (Gripenberg, 1952). The most frequently tree parts used for extract preparation were: wood (*n* = 19), bark (*n* = 17), and needles (*n* = 16), followed by resin (*n* = 8), sawdust (*n* = 4), and others (i.e., shoots, cone, branches, buds; *n* = 7). Twenty-six studies (43%) did not mention the harvesting date, while 34 studies (57%) mentioned the period of harvesting or collection of the tree source. Twenty-six (43%) of 34 studies mentioned both month/season and year, while 8 mentioned only year or season or month. Eight studies (13%) did not mention the extractive solvent or the type of preparation of the used extracts in the study. Eleven studies (18%) did not mention the origin of the sample or its collection place, one sample was from non-European origin, and the remaining came from Europe (Table 1).

**TABLE 1:** General overview over the 60 included articles in the review.

Tree source	Extractive solvent	Collection/harvest period	Site of collection/harvest	References
Bark	CH_2_Cl_2_	nd	Sweden	Norin and Winell (1974)
Bark	MeOH	nd	nd	Matthews et al. (1997)
Bark	EtOAc	September, 2008	Belgium	Frederich et al. (2009)
Bark	Chemically standardized	nd	Austria	Sgorlon et al. (2012)
Bark	MeOH	September, 2012	Austria	Laireiter et al. (2014)
Bark	Water (hot)	March, 2012	Switzerland	Bianchi et al. (2015)
Bark	*n*-heptane, MeOH, MeOH:water	October, 2014	France	Hubert et al. (2016)
Bark	EtOH	2017	Italy	Baldan et al. (2017)
Bark	CH_2_Cl_2_, EtOAc, MeOH	2009 and 2010	Finland and northern and far eastern Russia	Mulholland et al. (2017)
Bark	MeOH, water	nd	Germany	Wagner et al. (2019)
Bark	EtOH:water	nd	nd	Sillero et al. (2020)
Bark, resin (oleoresin)	CH_2_Cl_2_, ethyl acetate, MeOH (bark), *n*-hexane (turpentine)	December, 2013	Switzerland (bark), Austria (turpentine)	Thuerig et al. (2018)
Bark, wood	*n*-hexane	August, 2014	Czech Republic	Salem et al. (2015b)
Bark, wood	MeOH	February, 2015	Czech Republic	Salem et al. (2016)
Bark, wood (heartwood)	Water (acidic) followed by diethyl ether adition (3x)	End of 2014	Czech Republic	Salem et al. (2015a)
Branches	nd	March, May, June, August, September, November 1976 and February 1977	France, Italy, Poland, Czech Republic	Lang (1989)
Branches	Hydrodistillation without solvent, followed by solubilization in *n*-hexane	nd	nd	Holm and Hiltunen (1997)
Buds	Glycerol/EtOH and water/glycerol/EtOH	February–April, 2018 and 2019	Italy	Turrini et al. (2020)
Cones	Acetone, EtOH, MeOH	July–October, 2018	Hungary	Hofmann et al. (2020)
Essential oil (needles)	nd	June	Finland	Mofikoya et al. (2020)
Essential oil (needles, wood)	nd	nd	France	Garcia et al. (2017)
Essential oil (needles, wood, bark)	Hydrodistillation without solvent, followed by solubilization in *n*-pentane	nd	Germany	Kubeczka and Schultze (1987)
Flower, cone	MeOH	June, 1990	Norway	Andersen (1992)
Leaves	*n*-butanol, water (cold)	Autumn	nd	King (1966)
Leaves	Acetone, EtOH	Spring	nd	Goad and Goodwin (1967)
Leaves, branches, stem, root	nd	November, 1981	Germany	Lang and Messerer (1987)
Needles	Water	June–September	Austria	Lindner and Grill (1978)
Needles	EtOH	August, 1973	Netherlands	Niemann and Baas (1978)
Needles	Water	October, December, January	Netherlands	Kuiters and Sarink (1986)
Needles	*n*-hexane	July, November, December, 2003	Poland	Isidorov et al. (2005)
Needles	MeOH	September, 2010	Czech Republic	Malá et al. (2013)
Needles	nd	May, 2013	Switzerland	Churakova Sidorova et al. (2019)
Needles	Water	August, 2019	Poland	Dziedzinski et al. (2020)
Needles (wax)	CHCl_3_	July, 1985	Germany	Schulten et al. (1986)
Needles, shoots	Water (acidic)	May, July, October, 2011	Romania	Radulescu et al. (2013)
Needles, twigs, bark, wood, trunk	Hexane, MeOH, water	January–March, 2018	Switzerland	Piccand et al. (2019)
Oleoresin	Water (alkaline)	July, 1985	Ukraine	Bol’shakova et al. (1987)
Oleoresin	Diethyl ether, water (alkaline)	July, 1985	Ukraine	Bol’shakova et al. (1988)
Resin (callus resin, oleoresin)	EtOH	2003–2007	Finland	Holmbom et al. (2008)
Resin (oleoresin)	CH_2_Cl_2_	nd	nd	Norin (1972)
Resin (oleoresin)	Ether	nd	Austria, England	Mills (1973)
Resin, turpentine, essential oil	DMSO	nd	Germany	Urban et al. (2016)
Sawdust	EtOH, water	nd	nd	Farinacci et al. (2008)
Sawdust	EtOH*, n*-heptane, water	nd	Austria	Pferschy-Wenzig et al. (2008)
Sawdust	Chemically standardized	nd	Austria	Tedesco et al. (2015)
Sawdust	EtOH:water	November 2016–March 2017	Austria	Hochegger et al. (2019)
Turpentine	nd	nd	Austria	Dietemann et al. (2019)
Wood	Ether	nd	nd	Weinges (1961)
Wood	MeOH, water	nd	New Zeland	Uprichard (1963)
Wood	Ethyl acetate	August, 2015	France	Fu et al. (2018)
Wood	EtOH:toluene	nd	nd	Mecca et al. (2018)
Wood	Acetone, hexane	nd	Austria	Wagner et al. (2020)
Wood	*n*-hexane	nd	Czech Republic	Bajer et al. (2020)
Wood (heartwood)	Acetone	nd	nd	Gripenberg (1952)
Wood (heartwood, sapwood)	nd	May, 2003	France	Wajs et al. (2007)
Wood (knotwood)	Hexane, acetone:water	nd	Finland	Willför et al. (2003)
Wood (knotwood)	Hexane	nd	nd	Välimaa et al. (2007)
Wood (sapwood, heartwood)	Hexane	nd	Finland	Willför et al. (2005)
Wood (sawdust)	nd	nd	Austria	Becker et al. (2010)
Wood (softwood)	EtOH	nd	Poland	Kopania et al. (2012)

CHCl_3_, hloroform; CH_2_Cl_2_, dichloromethane; EtOAc, ethyl acetate; EtOH, ethanol; MeOH, methanol; nd, not declared.

**FIGURE 3:**
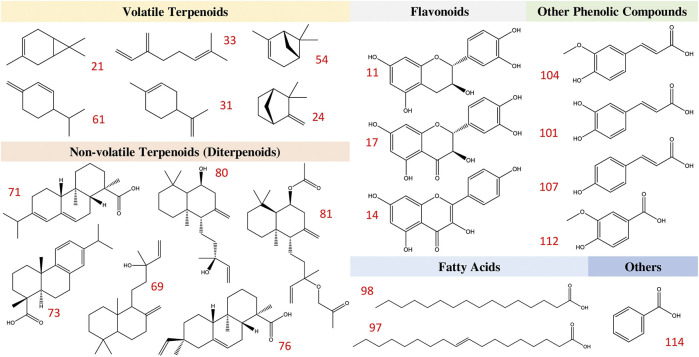
Phytochemical classes and their selected compounds identified in *L. decidua* Mill [Pinaceae].

Table 2 shows the compounds that were described in at least two publications and/or those found in at least two different parts of the tree. To better show the chemical variety presented in *L. decidua*, substances were categorized for different parts of the tree, the bark (*n* = 11), the needles (*n* = 19), the resin (*n* = 7) and the wood (*n* = 19). A total of 478 compounds were described for this tree (Supplementary Material), 118 are shown in Table 2. They were separated into different phytochemical categories, which included hydrocarbonates (**1**), carbohydrates (**2–9**), flavonoids (**10–18**), terpenoids and their derivatives (**19–90**), fatty acids (**91–100**), other phenolic compounds (**101–112**), and others classes (**113–118**). Terpenoids and their derivatives were among the most common/most important class of compounds described for *L. decidua*. Terpenoids and their derivatives in *L. decidua* were composed of volatile terpenoids (mainly mono and sesquiterpenes) and non-volatile terpenoids (diterpenoids), depending on the part of the plant being investigated. The resin contains mainly diterpenoids and phenolic compounds, whilst the wood, needles, and bark present a more varied chemical composition. The most often described compounds in each class were: carbohydrates—galactose (**4**), glucose (**6**); flavonoids—kaempferol (**14**), taxifolin (**17**); volatile terpenoids and their derivatives—3-carene (**21**), camphene (**24**), limonene (**31**), α/β-pinene (**54/62**), β-phellandrene (**61**); non-volatile terpenoids (diterpenoids)—13-epimanool (**69**), abietic acid (**71**), dehydroabietic acid (**74**), larixol (**80**), larixyl acetate (**81**); fatty acids—oleic acid (**97**), palmitic acid (**98**); phenolic acids—caffeic acid (**101**), ferulic acid (**104**), *p*-coumaric acid (**107**); others—benzoic acid (**114**). The chemical structures of the 22 most often described compounds are shown in Figure 3.

**TABLE 2:** Chemical data of the 118 most important identified compounds from *Larix decidua* Mill. [Pinaceae], organized by chemical class, tree part, identification and analytical method. Abbreviations described in Section 3.2.

Class	No	Compound	Tree part	Identification and analytical method	References
Hydrocarbonates	1	Methyl-cyclohexane	Bark, wood	GC-MS	Salem et al. (2015b)
Carbohydrates	2	Arabinose	Bark, wood	HPLC-UV, MALDI-TOF MS, GC-FID, GC-MS, GC, ATR-FTIR, NMR ^1^H	Willför et al. (2005); Bianchi et al. (2015); Hochegger et al. (2019)
	3	Fructose	Bark, needle	HPLC-UV, MALDI-TOF MS, GC, GC-MS	Isidorov et al. (2005); Bianchi et al. (2015)
	4	Galactose	Bark, needle, wood	HPLC-UV, MALDI-TOF MS, GC-FID, GC-MS, GC, ATR-FTIR, NMR ^1^H	Isidorov et al. (2005); Willför et al. (2005); Bianchi et al. (2015); Hochegger et al. (2019)
	5	Galacturonic acid	Bark, wood	HPLC-UV, MALDI-TOF MS, GC	Willför et al. (2005); Bianchi et al. (2015)
	6	Glucose	Bark, needle, wood	HPLC-UV, MALDI-TOF MS, HPLC, GC-FID, GC-MS, GC, ATR-FTIR, NMR ^1^H	Willför et al. (2005); Bianchi et al. (2015); Churakova Sidorova et al. (2019); Hochegger et al. (2019)
	7	Mannose	Bark, wood	HPLC-UV, MALDI-TOF MS, GC-FID, GC-MS, GC, ATR-FTIR, NMR ^1^H	Willför et al. (2005); Bianchi et al. (2015); Hochegger et al. (2019)
	8	Sucrose	Bark	HPLC-UV, MALDI-TOF MS, HPLC	Bianchi et al. (2015); Churakova Sidorova et al. (2019)
	9	Xylose	Wood	GC-FID, GC-MS, GC, ATR-FTIR, NMR ^1^H	Willför et al. (2005); Hochegger et al. (2019)
Flavonoids	10	Apigenin	Needle	UPLC, UV, TLC	Niemann and Baas, (1978); Dziedzinski et al. (2020)
	11	Catechin	Bark, needle	HPLC-DAD-MS, HPLC-DAD, HPLC-FLD-MS, UV-Vis	Baldan et al. (2017); Turrini et al. (2020)
	12	Dihydrokaempferol	Wood	TLC, GC-MS	Gripenberg (1952); Willför et al. (2003)
	13	Epicatechin	Bark, needle	HPLC-DAD-MS, HPLC-DAD, HPLC-FLD-MS, UV-Vis	Baldan et al. (2017); Turrini et al. (2020)
	14	Kaempferol	Needle, wood	UV, TLC, GC-MS, FT-RAMAN, FT-IR, FT-NIR, UPLC	Niemann and Baas (1978); Dziedzinski et al. (2020); Wagner et al. (2020)
	15	Luteolin	Bark, needle	HPLC-DAD-MS, HPLC-FLD-MS, UPLC	Baldan et al. (2017); Dziedzinski et al. (2020)
	16	Quercetin	Needle	HPLC-DAD, UV-Vis, UPLC	Dziedzinski et al. (2020); Turrini et al. (2020)
	17	Taxifolin	Bark, wood	GC-MS, FT-RAMAN, FT-IR, FT-NIR, TLC	Gripenberg (1952); Norin (1972); Wagner et al. (2019); Wagner et al. (2020)
	18	Vitexin	Needle	UV, TLC, UPLC	Niemann and Baas (1978); Dziedzinski et al. (2020)
Volatile Terpenoids	19	(E/Z)-*β*-farnesene	Needle, wood	GC-FID, GC-MS, NMR	Wajs et al. (2007); Garcia et al. (2017)
	20	1,8-cineole	Bark, needle	GC-FID, GC-MS	Kubeczka and Schultze (1987)
	21	3-carene	Bark, needle	GC-MS, GC-FID, GC, NMR	Kubeczka and Schultze (1987); Lang and Messerer (1987); Lang (1989); Holm and Hiltunen (1997); Isidorov et al. (2005); Garcia et al. (2017)
	22	4-terpinenol	Wood	GC-FID, GC-MS, NMR	Kubeczka and Schultze (1987); Salem et al. (2015b); Garcia et al. (2017)
	23	Bornyl acetate	Bark, needle, wood	GC-FID, GC-MS, GC, NMR	Kubeczka and Schultze (1987); Isidorov et al. (2005); Wajs et al. (2007); Garcia et al. (2017)
	24	Camphene	Bark, needle, wood	GC-MS, GC-FID, NMR	Kubeczka and Schultze (1987); Lang and Messerer (1987); Lang (1989); Holm and Hiltunen (1997); Wajs et al. (2007); Salem et al. (2015b); Garcia et al. (2017)
	25	Caryophyllene oxide	Bark, needle, wood	FT-ICR ESI/APPI, GC, GC-FID, GC-MS, NMR	Kubeczka and Schultze (1987); Isidorov et al. (2005); Garcia et al. (2017); Mofikoya et al. (2020)
	26	Cycloartenol	Needle, wood	GC-FID, HPLC-Q-ToF-MS	Goad and Goodwin (1967); Fu et al. (2018)
	27	Fenchol	Needle, wood	GC-FID, GC-MS, NMR	Salem et al. (2015b); Garcia et al. (2017)
	28	(Germacra-110)E,5E-dien-4-ol	Needle, wood	GC-FID, GC-MS	Kubeczka and Schultze (1987)
	29	Germacrene B	Bark, needle, wood	GC-FID, GC-MS	Kubeczka and Schultze (1987); Wajs et al. (2007)
	30	Germacrene D	Bark, needle, wood	FT-ICR ESI/APPI, GC-FID, GC-MS, NMR	Kubeczka and Schultze (1987); Wajs et al. (2007); Garcia et al. (2017); Mofikoya et al. (2020)
	31	Limonene	Bark, needle, wood	GC-FID, GC-MS, GC, NMR	Kubeczka and Schultze (1987); Lang and Messerer (1987); Holm and Hiltunen (1997); Isidorov et al. (2005); Wajs et al. (2007); Salem et al. (2015b); Garcia et al. (2017)
	32	Methyl thymol	Needle, wood	FT-ICR ESI/APPI, GC-FID, GC-MS	Wajs et al. (2007); Mofikoya et al. (2020)
	33	Myrcene	Bark, needle, wood	GC-FID, GC-MS, NMR	Kubeczka and Schultze (1987); Lang and Messerer (1987); Holm and Hiltunen (1997); Wajs et al. (2007); Garcia et al. (2017)
	34	Myrtenal	Bark, wood	GC-FID, GC-MS, NMR	Kubeczka and Schultze (1987); Garcia et al. (2017)
	35	Myrtenol	Bark, wood	GC-FID, GC-MS, NMR	Kubeczka and Schultze (1987); Garcia et al. (2017)
	36	*p*-cymen-8-ol	Needle, wood	GC-FID, GC-MS, NMR	Garcia et al. (2017)
	37	*p*-cymene	Bark, needle, wood	GC-FID, GC-MS, NMR	Kubeczka and Schultze (1987); Lang and Messerer (1987); Holm and Hiltunen (1997); Wajs et al. (2007); Garcia et al. (2017)
	38	Pinocarvone	Bark, wood	GC-FID, GC-MS, NMR	Kubeczka and Schultze (1987); Garcia et al. (2017)
	39	Sabinene	Bark, needle, wood	GC-FID, GC-MS	Kubeczka and Schultze (1987); Holm and Hiltunen (1997)
	40	T-cadinol	Bark, needle, wood	GC-FID, GC-MS, NMR	Kubeczka and Schultze (1987); Wajs et al. (2007); Garcia et al. (2017); Bajer et al. (2020)
	41	Terpinen-4-ol	Bark, needle	FT-ICR ESI/APPI, GC-FID, GC-MS	Kubeczka and Schultze (1987); Mofikoya et al. (2020)
	42	Terpinolene	Bark, needle, wood	GC-FID, GC-MS, NMR	Kubeczka and Schultze (1987); Lang and Messerer (1987); Holm and Hiltunen (1997); Wajs et al. (2007); Garcia et al. (2017)
	43	Thymol methyl ether	Needle, wood	FT-ICR ESI/APPI, GC-FID, GC-MS, NMR	Garcia et al. (2017); Mofikoya et al. (2020)
	44	T-muurolol	Bark, needle, wood	GC-FID, GC-MS, NMR	Kubeczka and Schultze (1987); Garcia et al. (2017)
	45	*Trans*-pinocarveol	Needle, wood	GC-FID, GC-MS, NMR	Garcia et al. (2017)
	46	*Trans*-verbenol	Bark, wood	GC-FID, GC-MS, NMR	Kubeczka and Schultze (1987); Garcia et al. (2017)
	47	Trieyelene	Bark, needle, wood	GC-FID, GC-MS	Kubeczka and Schultze (1987)
	48	Verbenene	Bark, needle	GC-FID, GC-MS, FT-ICR ESI/APPI	Kubeczka and Schultze (1987); Mofikoya et al. (2020)
	49	Verbenone	Needle, wood	GC-FID, GC-MS, NMR, FT-ICR ESI/APPI	Salem et al. (2015b); Garcia et al. (2017); Mofikoya et al. (2020)
	50	*α*-cadinol	Bark, needle, wood	GC-FID, GC-MS, NMR	Kubeczka and Schultze (1987); Garcia et al. (2017)
	51	*α*-humulene	Bark, needle, wood	GC-FID, GC-MS, NMR	Kubeczka and Schultze (1987); Garcia et al. (2017)
	52	*α*-muurolene	Needle, wood	GC-FID, GC-MS, NMR	Kubeczka and Schultze (1987); Wajs et al. (2007); Garcia et al. (2017); Bajer et al. (2020)
	53	*α*-phellandrene	Bark, needle, wood	GC-FID, GC-MS, NMR	Kubeczka and Schultze (1987); Holm and Hiltunen (1997); Wajs et al. (2007); Garcia et al. (2017)
	54	*α*-pinene	Bark, needle, wood	GC-FID, GC-MS, GC, NMR	Kubeczka and Schultze (1987); Lang and Messerer (1987); Holm and Hiltunen (1997); Isidorov et al. (2005); Wajs et al. (2007); Garcia et al. (2017)
	55	*α*-terpinene	Bark, needle, wood	GC-FID, GC-MS	Kubeczka and Schultze (1987); Lang and Messerer (1987); Holm and Hiltunen (1997); Wajs et al. (2007)
	56	*α*-terpineol	Bark, needle, wood	GC-FID, GC-MS, NMR	Kubeczka and Schultze (1987); Salem et al. (2015b); Garcia et al. (2017)
	57	*α*-terpinyl acetate	Bark, wood	GC-FID, GC-MS	Kubeczka and Schultze (1987); Wajs et al. (2007)
	58	*α*-thujene	Needle, wood	GC-MS	Holm and Hiltunen (1997); Salem et al. (2015b)
	59	*β*-carophyllene	Bark, needle, wood	GC-FID, GC-MS, GC, NMR	Kubeczka and Schultze (1987); Isidorov et al. (2005); Wajs et al. (2007); Garcia et al. (2017)
	60	*β*-elemene	Needle, wood	GC-FID, GC-MS, NMR	Wajs et al. (2007); Garcia et al. (2017)
	61	*β*-phellandrene	Bark, needle, wood	GC-FID, GC-MS, GC, NMR	Kubeczka and Schultze (1987); Lang and Messerer (1987); Holm and Hiltunen (1997); Isidorov et al. (2005); Wajs et al. (2007); Garcia et al. (2017)
	62	*β*-pinene	Bark, needle, wood	GC-FID, GC-MS, GC, NMR	Kubeczka and Schultze (1987); Lang and Messerer (1987); Holm and Hiltunen (1997); Isidorov et al. (2005); Wajs et al. (2007); Garcia et al. (2017)
	63	*γ*-cadinene	Bark, needle, wood	GC-FID, GC-MS, NMR	Kubeczka and Schultze (1987); Garcia et al. (2017)
	64	*γ*-muurolene	Needle	GC-FID, GC-MS, GC, NMR	Isidorov et al. (2005); Garcia et al. (2017)
	65	*γ*-terpinene	Bark, needle, wood	GC-FID, GC-MS	Kubeczka and Schultze (1987); Holm and Hiltunen (1997); Wajs et al. (2007)
	66	*δ*-3-carene	Wood	GC-FID, GC-MS	Kubeczka and Schultze (1987); Wajs et al. (2007)
	67	*δ*-cadiene	Bark, needle	GC-FID, GC-MS	Kubeczka and Schultze (1987)
	68	*δ*-cadinene	Needle, wood	GC-FID, GC-MS, NMR	Kubeczka and Schultze (1987); Wajs et al. (2007); Garcia et al. (2017); Bajer et al. (2020)
Non-Volatile Terpenoids (Diterpenoids)	69	13-epimanool	Bark, resin, wood	IR, ^1^H/^13^C NMR, TLC, UV-Vis, GC, GC-FID, GC-MS	Norin (1972); Mills (1973); Norin and Winell (1974); Bol’shakova et al. (1988); Salem et al. (2015b); Thuerig et al. (2018); Dietemann et al. (2019)
	70	Abietadiene	Needle, wood	FT-ICR ESI/APPI, GC-FID, GC-MS, NMR	Garcia et al. (2017); Mofikoya et al. (2020)
	71	Abietic acid	Bark, needle, resin, wood	IR, UV-Vis, NMR, GC, GC-FID, GC-MS, FT-ICR ESI/APPI, HPLC-Q-ToF-MS	Mills (1973); Bol’shakova et al. (1987); Isidorov et al. (2005); Holmbom et al. (2008); Pferschy-Wenzig et al. (2008); Salem et al. (2016); Fu et al. (2018); Dietemann et al. (2019); Mofikoya et al. (2020)
	72	Abietol	Resin	GC-FID, GC-MS	Mills (1973); Holmbom et al. (2008)
	73	Dehydroabietic acid	Needle, resin, wood	IR, UV-Vis, NMR, GC, GC-FID, GC-MS, FT-ICR ESI/APPI	Mills (1973); Bol’shakova et al. (1987); Isidorov et al. (2005); Holmbom et al. (2008); Pferschy-Wenzig et al. (2008); Dietemann et al. (2019); Mofikoya et al. (2020)
	74	Dehydroabietol	Resin, wood	GC-FID, GC-MS	Mills (1973); Pferschy-Wenzig et al. (2008)
	75	Isopimaral	Needle, wood	GC-FID, GC-MS, NMR	Garcia et al. (2017)
	76	Isopimaric acid	Resin, wood	IR, UV-Vis, NMR, GC, GC-FID, GC-MS, HPLC-Q-ToF-MS	Mills (1973); Bol’shakova et al. (1987); Holmbom et al. (2008); Pferschy-Wenzig et al. (2008); Fu et al. (2018); Dietemann et al. (2019)
	77	Isopimarinal	Resin, wood	UV-Vis, GC, GC-FID, GC-MS	Bol’shakova et al. (1988); Bajer et al. (2020)
	78	Isopimarol	Needle, wood	FT-ICR ESI/APPI, GC-FID, GC-MS	Bajer et al. (2020); Mofikoya et al. (2020)
	79	Lariciresinol	Resin, wood	GC-FID, GC-MS	Willför et al. (2003); Holmbom et al. (2008)
	80	Larixol	Bark, resin, wood	UV-Vis, GC, GC-FID, GC-MS, FT-RAMAN, FT-IR, FT-NIR, ^1^H/^13^C NMR	Norin (1972); Mills (1973); Bol’shakova et al. (1988); Salem et al. (2015b); Thuerig et al. (2018); Dietemann et al. (2019); Wagner et al. (2020)
	81	Larixyl acetate	Bark, resin, wood	UV-Vis, GC, GC-FID, GC-MS, 1H/13C NMR, ESIMS, IR	Norin (1972); Mills (1973); Bol’shakova et al. (1988); Pferschy-Wenzig et al. (2008); Mulholland et al. (2017); Thuerig et al. (2018); Dietemann et al. (2019)
	82	Levopimaric acid	Resin	IR, UV-Vis, NMR, GC, GC-FID, GC-MS	Mills (1973); Bol’shakova et al. (1987); Holmbom et al. (2008)
	83	Manool	Needle, wood	GC-FID, GC-MS, NMR	Wajs et al. (2007); Garcia et al. (2017)
	84	Neoabietic acid	Resin	IR, UV-Vis, NMR, GC, GC-FID, GC-MS	Mills (1973); Bol’shakova et al. (1987); Holmbom et al. (2008); Dietemann et al. (2019)
	85	Palustric acid	Resin, wood	IR, UV-Vis, NMR, GC, GC-FID, GC-MS	Mills (1973); Bol’shakova et al. (1987); Holmbom et al. (2008); Pferschy-Wenzig et al. (2008); Dietemann et al. (2019)
	86	Palustrol	Resin	UV-Vis, GC, GC-FID, GC-MS	Bol’shakova et al. (1988); Holmbom et al. (2008)
	87	Pimarate	Resin	GC-FID, GC-MS	Mills (1973); Dietemann et al. (2019)
	88	Pimaric acid	Needle, Resin	FT-ICR ESI/APPI, GC, GC-MS, GC-FID	Isidorov et al. (2005); Holmbom et al. (2008); Mofikoya et al. (2020)
	89	Sandaracopimaric acid	Resin	IR, UV-Vis, NMR, GC, GC-FID, GC-MS	Mills (1973); Bol’shakova et al. (1987); Holmbom et al. (2008); Dietemann et al. (2019)
	90	Secoisolariciresinol	Resin, wood	GC-FID, GC-MS	Willför et al. (2003); Holmbom et al. (2008)
Fatty acids	91	Arachidic acid	Bark, wood	GC	Salem et al. (2015a)
	92	Hexadecanoic acid	Needle, wood	GC, GC-MS	Isidorov et al. (2005); Mecca et al. (2018)
	93	Linoleic acid	Bark, wood	IR, NMR, TLC, HPLC-Q-ToF-MS	Norin and Winell, (1974); Fu et al. (2018)
	94	Margarinic acid	Bark, wood	GC	Salem et al. (2015a)
	95	Myristic acid	Bark	IR, NMR, TLC, GC	Norin and Winell, (1974); Salem et al. (2015a)
	96	Octadecanoic acid	Needle, wood	GC, GC-MS	Isidorov et al. (2005); Mecca et al. (2018)
	97	Oleic acid	Bark, needle, wood	FT-ICR ESI/APPI, GC, GC-MS, IR, NMR, TLC	Norin and Winell (1974); Isidorov et al. (2005); Mecca et al. (2018); Mofikoya et al. (2020)
	98	Palmitic acid	Bark, needle, wood	IR, NMR, TLC, GC, FT-ICR ESI/APPI, HPLC-Q-ToF-MS	Norin and Winell (1974); Salem et al. (2015a); Fu et al. (2018); Mofikoya et al. (2020)
	99	Pentadecanoic acid	Bark, wood	GC, GC-MS	Salem et al. (2015a); Mecca et al. (2018)
	100	Stearic acid	Bark, needle, wood	IR, NMR, TLC, GC, FT-ICR ESI/APPI	Norin and Winell, (1974); Salem et al. (2015a); Mofikoya et al. (2020)
Other phenolic compounds	101	Caffeic acid	Needle, resin	HPLC-DAD, UV-Vis, GC-FID, GC, GC-MS, HPLC, UPLC	Lindner and Grill (1978); Kuiters and Sarink (1986); Holmbom et al. (2008); Malá et al. (2013); Dziedzinski et al. (2020); Turrini et al. (2020)
	102	Chlorogenic acid	Needle	GC, HPLC, UPLC	Lindner and Grill (1978); Malá et al. (2013); Dziedzinski et al. (2020)
	103	Cinnamic acid	Needle	GC-FID, GC, UPLC	Lindner and Grill (1978); Kuiters and Sarink, (1986); Dziedzinski et al. (2020)
	104	Ferulic acid	Needle, resin	GC-FID, GC-MS, GC, HPLC, UV, TLC, UPLC	Lindner and Grill (1978); Niemann and Baas (1978); Kuiters and Sarink, (1986); Holmbom et al. (2008); Malá et al. (2013); Dziedzinski et al. (2020)
	105	Gallic acid	Needle	GC-FID, GC, HPLC, UPLC	Lindner and Grill (1978); Kuiters and Sarink, (1986); Malá et al. (2013); Dziedzinski et al. (2020)
	106	Lariciresinol	Bark	GC-MS, NMR, ESIMS, IR	Mulholland et al. (2017); Wagner et al. (2019)
	107	*p*-coumaric acid	Needle, resin	GC-FID, GC-MS, GC, HPLC, UV, TLC, UPLC	Lindner and Grill (1978); Niemann and Baas (1978); Kuiters and Sarink (1986); Holmbom et al. (2008); Malá et al. (2013); Dziedzinski et al. (2020)
	108	*p*-hydroxy benzoic acid	Needle	GC-FID, HPLC, UV, TLC, UPLC	Niemann and Baas (1978); Kuiters and Sarink (1986); Malá et al. (2013); Dziedzinski et al. (2020)
	109	Pinoresinol	Bark, resin	GC-FID, GC-MS	Holmbom et al. (2008); Wagner et al. (2019)
	110	Protocatechuic acid	Needle	HPLC, GC	Lindner and Grill (1978); Malá et al. (2013)
	111	Syringic acid	Needle	GC-FID, GC, UPLC	Lindner and Grill (1978); Kuiters and Sarink, (1986); Dziedzinski et al. (2020)
	112	vanillic acid	Needle	GC-FID, GC, HPLC, UV, TLC, UPLC	Lindner and Grill (1978); Niemann and Baas, (1978); Kuiters and Sarink, (1986); Malá et al. (2013); Dziedzinski et al. (2020)
Other compounds	113	Ascorbic acid	Needle	GC, HPLC	Lindner and Grill (1978); Radulescu et al. (2013)
	114	Benzoic acid	Needle	FT-ICR ESI/APPI, GC-FID, GC	Lindner and Grill (1978); Kuiters and Sarink, (1986); Mofikoya et al. (2020)
	115	Citric acid	Needle	GC, GC-MS	Lindner and Grill (1978); Isidorov et al. (2005)
	116	Quinic acid	Needle	FT-ICR ESI/APPI, GC	Lindner and Grill (1978); Mofikoya et al. (2020)
	117	Salicylic acid	Needle	GC-FID, UPLC	Kuiters and Sarink (1986); Dziedzinski et al. (2020)
	118	Succinic acid	Needle	GC, GC-MS	Lindner and Grill (1978); Isidorov et al. (2005)

Different analytical methodologies were used for the separation, isolation, structural elucidation or identification of these compounds, such as TLC (thin layer chromatography), HPLC (high performance liquid chromatography), GC (gas chromatography), NMR (nuclear magnetic resonance), FTIR (Fourier transform infrared spectroscopy), among others. The most often used technique was GC, coupled to a flame ionization (FID) or a mass spectrometer (MS) as detectors, for the identification of terpenoids and their derivatives, fatty acids and phenolic compounds. For the identification of flavonoids, liquid chromatography (LC) techniques were the most often used, such as HPLC and ultra-performance liquid chromatography (UPLC). The identification/structural elucidation of carbohydrates presented a wider variety of techniques, using LC, GC, as well as NMR, matrix-assisted laser desorption/ionization-time of flight (MALDI-TOF) and FTIR—attenuated total reflectance (ATR-FTIR) techniques.

### 3.3 Biological *In Vitro* Studies

#### 3.3.1 Antimicrobial Activity

Antibacterial effect of bark and wood discs, as well as their methanol extracts were tested against four different species of bacteria (Table 3) (Laireiter et al., 2014). Larch bark discs inhibited *S. aureus* growth, whilst the wood discs did not. The wood discs methanol extract did not show any inhibitory effect on *S. aureus*, in contrast to the bark sawdust methanol extract. The bark discs and extract presented inhibitory effects on *S. aureus* while wood discs and extracts did not, showing that the tree source is an important factor for biological effects of *L. decidua* (Laireiter et al., 2014). Välimaa et al. (2007) evaluated the antimicrobial properties against bacteria and fungi (Table 3) of a hexane wood extract, followed by extraction with acetone/water (95:1 *v*/*v*), which showed an inhibition against *S. infantis* (11%), *B. cereus* (31%), *C. albicans* (32%) and *S. cerevisiae* (17%). Bark methanol and aqueous extracts were tested against 4 species of microorganisms (Table 3), by which only the methanol extract affected the growth of *S. aureus* with an inhibitory zone of 8.2 mm (Wagner et al., 2019). The authors attributed the activity to the presence of the flavonoid kaempferol and the stilbenoid astringin (Wagner et al., 2019). Three different bark extracts (*n*-heptane, methanol, and methanol/water 50:50 (*v*/*v*)) were tested against *S. aureus*, in which the methanol (++++), methanol/water (+++), and *n*-heptane (+) presented antibacterial activity in a descending way, respectively (Hubert et al., 2016). The activity was correlated to the presence of phenolic compounds (Hubert et al., 2016). These studies showed that the antimicrobial activity [minimum inhibitory concentration (MIC) and minimum fungicidal concentration (MFC)] depends on the plant part used and the solvent, as shown by the different effects on several microorganisms (Salem et al., 2016). Wood and bark methanol extracts were tested against nine different bacteria and six different fungi (Table 3). The bark extract presented lower MIC (0.11 mg/ml) compared to that of wood (0.13 mg/ml), in addition the minimum bactericidal concentration (MBC) varied from 0.36–0.96 mg/ml and 0.33–1.1 mg/ml, for the bark and wood extracts, respectively (Salem et al., 2016). All cited studies showed a better antimicrobial activity when using bark extracts when compared to wood. Two studies evaluated the activity of different larch extracts and isolated compounds against the fungus *Plasmopara viticola*. The MIC to completely inhibit zoospore germination and/or activity of *P. viticola* was 23 μg/ml for a turpentine formulation, 6 and 14 μg/ml for larixyl acetate and larixol, respectively (Thuerig et al., 2018). The authors suggest that both compounds represent valid candidates for use as antifungal substances in organic vineyards thereby reducing the use of copper. Bark CH_2_Cl_2_ extract (1 mg/ml) presented high efficacy and the isolated compounds (larixol, larixyl acetate and lariciresinol) at the same concentration (1.0 mg/ml) were very efficient (between 90% and 100%) against grapevine downy mildew, whereby larixyl acetate was the most efficient, showing 70% of efficacy at 0.1 mg/ml. This was the first report concerning the activity of larch extracts against plant pathogenic oomycetes, which counts as a renewable resource at low prices for a sustainable plant protection (Mulholland et al., 2017). Water extract of needles presented antimicrobial activity against microorganisms of Gram-positive and Gram-negative bacteria as well as mold and yeast, with the most prominent result for *L. fermentum, S. aureus, C. butyricum and B. coagulans* (inhibition zones of 13 ± 2, 11 ± 2, and 10 ± 1, 10 ± 2 mm, respectively), which was correlated with the presence of phenolic compounds (Dziedzinski et al., 2020).

**TABLE 3:** Biological *in vitro* studies with *Larix decidua* Mill. [Pinaceae].

Type of investigation	Sample	Assay	Cell/microorganism/material	Results	Author
Antimicrobial	MeOH bark and wood extracts	Agar-diffusion test	*S. aureus*, *P. aeruginosa*, *E. faecium*, *B. subtilis*	Larch bark discs inhibited the growth of *S. aureus,* as well as bark sawdust MeOH extract (25 and 50 µL). In contrast, wood discs and wood MeOH extract did not present any inhibitory activity. Concluded that bark compounds are responsible for the antimicrobial activity	Laireiter et al. (2014)
	MeOH and water bark extract	Agar diffusion test	*S. aureus, E. coli, P. aeruginosa, C. albicans*	MeOH extract (25/50 µL) presented antimicrobial effect against *S. aureus* (8.2 mm)	Wagner et al. (2019)
	*n*-heptane, MeOH, MeOH:water bark extracts	Immersion bioautography method	*S. aureus*	MeOH and MeOH:water extracts displayed antibacterial activity	Hubert et al. (2016)
	MeOH bark and wood extracts	Antifungal activity by the microdilution method and spore suspension; antibacterial activity by the micro-dilution method	*P. funiculosum, P. ochrochloron, A. niger, A. flavus, A. ochraceus, C. albicans, B. cereus, D. solani, E. coli, L. monocytogenes, M. flavus, P. aeruginosa, P. atrosepticum, P. carotovorum ssp. carotovorum, S. aureus*	MIC and MFC values of wood extracts were higher than the bark. Wood extract showed the highest MIC and MFC for *A. flavus, A. niger, P. funiculosum.* Wood and bark extracts exhibited antibacterial activity against all bacteria, but the bark was higher [MIC (0.11–0.54 mg/ml) and MBC (0.36–0.96 mg/ml)] than the wood one [MIC (0.13–0.54 mg/ml) and MBC (0.33–1.1 mg/ml)]	Salem et al. (2016)
	Hexane wood sawdust	Growth inhibition test using broth subcultures; inhibition zones in fungal confluent growth	*E. coli, S. infantis, P. fluorescens, B. cereus, S. aureus, L. monocytogenes, L. plantarum, C. albicans, S. cerevisae*	4 µL of extract (10 mg extractives/mL) presented inhibitory effect against *S. infantis* (11%), *B. cereus* (31%), *C. albicans* (32%) and *S. cerevisiae* (17%)	Välimaa et al. (2007)
	Turpentine, isolated compounds	Antifungal inhibition germination and/or activity of zoospores (MIC_100_)	*Plasmopara viticola*	Larch turpentine extract presented MIC_100_ of 23 μg/ml, larixyl acetate 6 μg/ml, and larixol 14 μg/ml	Thuerig et al. (2018)
	CH_2_Cl_2_ bark extract, isolated compounds	Antifungal inhibition germination and/or activity of zoospores	*Plasmopara viticola*	CH_2_Cl_2_ extract (1 mg/ml) showed very high efficacies between 80% and 98% against downy mildew. Larixol, larixyl acetate and lariciresinol at 1 mg/ml presented efficacies between 90% and 100%	Mulholland et al. (2017)
	Water needle extract	Antibacterial and antifungal activity through growth inhibition zone	*K. penumoniae, S. enteritidis, P. aeruginosa, A. baumannii, E. faecium, S. aureus, L. fermentum, C. butyricum, L. monocytogenes, B. coagulans, C. utilis, Aspergillus* spp*., Fusarium* spp*.*	Water extract (150 µL) presented antimicrobial activity against all microorganisms tested, with higher growth inhibition zone for gram-positive bacteria, such as *L. fermentum* (13 ± 2 mm) and *S. aureus* (11 ± 2 mm)	Dziedzinski et al. (2020)
Cytotoxicity	EtOAc bark extract	MTT assay	Human colon metastatic cell (LoVo), human prostate metastatic cell (PC3), human glioblastoma astrocytoma (U373)	It was observed no selectivity of the EtOAc extract on the tested cell lines: LoVo (IC_50_ 68 μg/ml), PC3 (IC_50_ 52 μg/ml), U373 (IC_50_ 56 μg/ml), but it presented interesting cytotoxicity	Frederich et al. (2009)
	Isolated compounds	MTT assay, PI assay	Human embryonic kidney (HEK)	Larixol and larixyl acetate did not affect cell viability and proliferation, after 10 min, but larixyl acetate decreased cell viability after 24 h after incubation (2.5–100 µM)	Urban et al. (2016)
Other	Turpentine, resin, essential oil, isolated compounds	Metabolic/physiological activity; TRPC inhibition by Ca^2+^ variation	Human embryonic kidney (HEK)	Larch turpentine and Venice Turpentine presented IC_50_ of 13 mg/L and 140 mg/L, respectively, over TRPC6 channel, and 300 mg/L, 110 mg/L, and 610 mg/L, respectively, over TRPC3 channel. Larixol and larixyl acetate blocked Ca^2+^ channels. Concluded that larch-derived labdane-type diterpenes are TRPC6-selective inhibitors	Urban et al. (2016)
	*n*-heptane, MeOH, MeOH:water bark extracts	Metabolic/physiological activity; elastase inhibitory assay; colagenase inhibitory assay; tyrosinase inhibitory assay	Porcine pancreatic elastase type IV; collagenase from *Clostridium histolyticum*; mushroom tyrosinase	MeOH extract (300 μg/ml) exhibited the highest elastase inhibitory activity (>80%), followed by the MeOH:water (300 μg/ml) extract (>70%). MeOH extract (150 μg/ml) exhibited the highest collagenase inhibitory activity (>90%), followed by the MeOH:water (150 μg/ml) extract (>80%). MeOH extract (300 μg/ml) exhibited the significant tyrosinase inhibitory activity (>50%), followed by the MeOH:water (300 μg/ml) extract (>40%)	Hubert et al. (2016)
	Wood sawdust	Blood/immune system activity; toxin receptor binding through antibody detection by ELISA	*E. coli* heat-labile enterotoxin (LTp-I)	Larch sawdust (50 mg/ml) reduced (61.8%–63.6%) toxin binding to GM1 (ganglioside natural receptor for cholera toxin). In addition, larch arabinogalactan at the same concentration reduced (15.2%–53.6%) toxin binding	Becker et al. (2010)
	*n*-heptane, EtOH, water sawdust extracts	Blood/immune system activity; COX-1, COX-2 and LTB4 inhibition assay	Purified ram seminal vesicles for COX-1, purified sheep placental cotyledons for COX-2, human polymorphonuclear leukocytes	*n*-heptane extract (20 μg/ml) possessed pronounced inhibitory activity, with IC_50_ values of 5 μg/ml, 0.1 μg/ml, and 11.1 μg/ml for COX-1, COX-2, and LTB4, respectively. The IC_50_ of the 70% EtOH extract against COX-2 was 0.8 μg/ml. In contrast to the extracts themselves, the isolated compounds were more active against LTB4 than to COX-2. Only larixyl acetate and palustric acid presented inhibitory activity against COX-2 (IC_50_ value of 95.1 and 57.9 µM, respectively). Larixol and abietic acid methyl ester were inactive, whilst larixyl acetate, isopimaric acid, palustric acid, dehydroabietic acid, dehydroabietinol, abietic acid, and abietinol were selectively inhibitors to LTB4 formation	Pferschy-Wenzig et al. (2008)

#### 3.3.2 Cytotoxicity

An ethyl acetate macerated bark extract was tested for its anticancer potential *in vitro*, against three different human cancer cell lines (PC3, U373, LoVo; Table 3). The crude extract was incubated for 72 h and the cell viability was evaluated by 3-[4,5-dimethylthiazol-2-yl]-2,5 diphenyl tetrazolium bromide (MTT). Human prostatic adenocarcinoma (PC3; IC_50_ 52 μg/ml) was slightly more sensitive to the extract than the human glioblastoma (U373; IC_50_ 56 μg/ml), and lastly human colorectal adenocarcinoma (LoVo; IC_50_ 68 μg/ml) was the most resistant (Frederich et al., 2009). However, other tree extracts (*C. betulus* [LoVo: IC_50_ 85 μg/ml], *C. sativa* [LoVo: IC_50_ 76 μg/ml; PC3: IC_50_ 96 μg/ml; U373: IC_50_ 86 μg/ml], *F. sylvatica* [PC3: IC_50_ 70 μg/ml], *I. aquifolium* [PC3: IC_50_ 76 μg/ml], *Q. petrea* [PC3: IC_50_ 69 μg/ml], *Q. robur* [LoVo: IC_50_ 80 μg/ml; PC3: IC_50_ 75 μg/ml], *R. pseudoacacia* [LoVo: IC_50_ 77 μg/ml; PC3: IC_50_ 69 μg/ml; U373: IC_50_ 94 μg/ml]) presented lower inhibitory activity against these human cancer cells (Frederich et al., 2009). Two isolated compounds from larch, larixol and larixyl acetate, were incubated with human embryonic kidney cells (HEK293). The integrity of the cells using propidium iodide (PI) assay (after 10 min and 24 h of compounds incubation) and their cell viability by MTT (after 24 h of compounds incubation) were evaluated (Urban et al., 2016). Membrane integrity was maintained at the three concentrations tested (2.5, 5, 10 µM) and cell viability and proliferation were also unaffected by the two tested compounds (25 and 50 µM) (Urban et al., 2016).

#### 3.3.3 Other *In Vitro* Assays

In order to investigate the activity of some natural products that could abrogate pathophysiological responses within pulmonary and renal diseases, Ca^2+^ measurement was assessed on HEK 293 cell line (Table 3) (Urban et al., 2016). Larch turpentine (IC_50_ 13 mg/L) and Venice Turpentine (IC_50_ 140 mg/L; a mixture of larch turpentine and colophony) blocked Ca^2+^ entry through TRPC6 channel in a dose dependent manner, whilst the larch essential oil presented no activity. The authors concluded that the biological activity is due to the presence of the non-volatile resiniferous compounds, larixol (IC_50_ 2.04 µM) and larixyl acetate (IC_50_ 0.58 µM) (Urban et al., 2016).

Investigation on different tree species extracts for their potential as dermo-cosmetics assayed the effect of three different extracts from larch bark on three skin enzymes: collagenase, elastase and tyrosinase (Table 3). The incubation period for the collagenase and tyrosinase assays was 10 min and for the elastase 30 min, and the concentration of the tested extracts varied for each assay, in a range of 60–300 μg/ml (Hubert et al., 2016). Methanol extract was the most potent, followed by the methanol:water (50:50 *v*/*v*), and the less active was the *n*-heptane extract, for all assays. Elastase inhibitory activity was higher than 80% and 70% for the methanol and methanol:water extracts (300 μg/ml), respectively. The same profile was observed for collagenase, in which the inhibitory activity was higher than 90% and 80% at 150 μg/ml. Tyrosinase inhibitory activity was lower, but presented 50% and 40% for the methanol and methanol:water extracts (300 μg/ml), respectively. These results showed the potential of the bark extract to keep the skin homeostasis, by avoiding degradation of skin proteins, and to slow down skin pigments production in melanocytes, mainly due to the presence of phenolic substances (Hubert et al., 2016).

Becker et al. (2010) investigated the competitive inhibition of GM1-binding sites for cholera enterotoxins (Table 3). Larch wood sawdust and arabinogalactan (isolated from larch wood) at 0.5, 5 and 50 mg/ml presented a dose-dependent inhibition of toxin binding to GM1. An interesting finding for the wood sawdust (50 mg/ml) was that even when the toxin was already bound to the receptor, it was able to inhibit (62%) the binding at the same proportion as the pre-treatment (64%) or the simultaneous application of extract and toxin (62%). In contrast, arabinogalactan added after the toxin was already bound presented a very low interfering effect (15%) (Becker et al., 2010).

The influence of larch sawdust extracts on arachidonic acid cascade, a pro-inflammatory pathway, was evaluated in order to discover bioactive constituents from food, pharmaceutical and agricultural industries’ waste (Table 3) (Pferschy-Wenzig et al., 2008). Water, ethanol 70% and *n*-heptane extracts were prepared and then lyophilized. For the experiments, the dried samples were dissolved in absolute ethanol at a final concentration of 20 μg/ml. The *n*-heptane extract possessed pronounced anti-inflammatory activity, followed by the ethanol 70% extract and the water extract. The IC_50_ values were 5 μg/ml, 0.1 μg/ml, and 11.1 μg/ml for COX-1, COX-2, and LTB_4_, respectively, for the *n*-heptane extract, while for the ethanol 70% extract it was 0.8 μg/ml for COX-2. To discover the active compounds, isolation of different chemicals from the *n*-heptane extract was carried out. The isolated diterpenes (Table 3) had inhibitory activity for LTB4, but only two presented inhibitory activity for COX-2, and none for COX-1. The authors inferred that other compounds than the isolated diterpenes must be responsible for the crude *n*-heptane extract inhibitory activity on COX-1 and COX-2, such as fatty acids, and that a series of diterpene acids were selective inhibitors of LTB4 (Pferschy-Wenzig et al., 2008).

### 3.4 *In Vivo* Studies

Two studies evaluated standardized larch sawdust as ruminants’ dietary complement in comparison to controls (Table 4) (Sgorlon et al., 2012; Tedesco et al., 2015). Investigation of supplementation in 24 dairy cows in mid-lactation evaluated the effects on blood parameters and milk composition (Tedesco et al., 2015). The manufacturer standardized it by its content in fibre, protein, fat, ash, and lignin, whilst the group evaluated it through HPLC, standardizing it as 0.7% of taxifolin and 0.7% of dihydrokaempferol. It was given at a concentration of 300 g/day/cow, for 20 days, twice a day, and the milk parameters were evaluated at days 0, 7, 14 and 20, while blood parameters were just measured at days 0 and 20. No effect on milk parameters was identified, in contrast to urea, bilirubin, cholesterol, and VLDL concentration, which decreased in the blood, suggesting liver improvement, probably due to the presence of taxifolin, a compound that acts like statins and has antioxidant activity, contributing to hepatoprotection (Tedesco et al., 2015). Taxifolin was described in the bark, wood and the resin, making them sources to obtain this promising compound (Gripenberg, 1952; Norin, 1972; Wagner et al., 2019; Wagner et al., 2020). Sgorlon et al. (2012) evaluated larch sawdust counteraction on gene expression in blood leukocytes after ACTH (adenocorticotropic hormone)-induced cortisol of thirty-six Sarda sheep. The amount of 50 g/head (5% of dry matter intake), which contained larixyl acetate and arabinogalactan as bioactive molecules, was given to the animals 15 days before treatment with ACTH. Cortisol concentration increased 8-fold for 3 and 51 h after ACTH treatment compared to the basal concentration, also increasing the down-regulation of transcripts up to 85.5% after 51 h. Larch sawdust supplementation regulated genes responsive to stress (GPX7, GADD45B, XRCC6, WRN1P1), to cell death pathways (NR4A1, GSK3B, TP53), to immune response (IFNG, MAPK3, NFkBIB) suggesting its use as an anti-inflammatory candidate for gene modulation (Sgorlon et al., 2012). The anti-inflammatory activity of larch sawdust was verified on sheep neutrophils (Farinacci et al., 2008) and against LTB4 and COX-2 formation (Pferschy-Wenzig et al., 2008), both studies in a different area of investigation but focused on biological anti-inflammatory activity.

**TABLE 4:** Studies about *in vivo* and *ex vivo* applications of *Larix decidua* Mill. [Pinaceae] derivatives.

Type of investigation	Investigation	Sample	Biological source/animal model	Assay	Results	Author
*In vivo*	Effect of larch sawdust supplementation on blood parameters and milk composition	Chemically standardized sawdust	24 multiparous Italian Friesian dairy cows in mid-lactation	300 g of milled sawdust/day/cow	Milk parameters were unaffected. Blood metabolites were affected by larch sawdust intake. Blood urea concentration decreased, tendency for lowering glucose, total bilirubin decreased, and cholesterol tended to be lower than control. Concluded that larch improves liver function	Tedesco et al. (2015)
	The effect of dietary administration on the modification of biological processes induced by high plasma cortisol	Chemically standardized sawdust	36 Sarda sheep	1 kg/head twice a day of basal diet, treating with 50 g/head of *L. decidua* Mill. [Pinaceae] bark 22 h before using twice a day with 0.5 ml of ACTH agonist (5 IM injections)	Cortisol concentration increased 8-fold greater than basal concentration (*p* < 0.001) with Larch use after ACTH treatment. After 51 h of ACTH and Larch bark treatment, down-regulation of transcripts increased (85.8%). Concluded that larch bark could be candidate as dietary supplements to modulate the modification of gene expression related to increased concentrations of cortisol	Sgorlon et al. (2012)
*Ex vivo*	Evaluate the immunomodulatory activity of waste extracts on ovine neutrophils	EtOH 70% and water sawdust extracts	Ovine neutrophils from 8 healthy sheep	MTT viability assay; acid phosphatase adhesion assay; superoxide production assay by horse-heart ferricytochrome c	EtOH 70% (2.23–60 μg/ml) extract significantly reduced the MTT metabolism of neutrophils in a dose-dependent manner (>60%), whilst the aqueous (6.67–180 μg/ml) had no effect on neutrophil viability. The EtOH extract strongly blocked neutrophil adhesion (IC50 10.89 μg/ml) and inhibited the superoxide production from activated neutrophils (IC50 8.15 μg/ml) in a dose-dependent manner. Concluded that extract has anti-inflammatory activity on sheep neutrophils, possibly due to the presence of flavonoids and arabinogalactan	Farinacci et al. (2008)

Unfortunately, abietic acid and abietanes are readily oxidized (Scalarone et al., 2002; Osete-Cortina and Domenech-Carbo, 2005). These oxidized products, such as 15-hydroperoxydehydroabietic acid, 15-hydroxyabietic acid methyl ester, 7-oxodehydroabietic acid methyl ester, are reported as responsible for contact allergy and dermatitis (Karlberg and Liden, 1985; Hausen et al., 1993; Downs and Sansom, 1999; Barchino-Ortiz et al., 2008). However, animal experiments could not substantiate this concern. *L. decidua* seems to be safe and well tolerated. In animal studies, oral intake of larch sawdust did not show any harm to cows or sheep (Tedesco et al., 2015). Each animal received 300 g of milled sawdust daily for 20 days, standardized with 0.7% of taxifolin and 0.7% of dihydrokaempferol. These two compounds are present in the bark (Wagner et al., 2019), resin (Norin, 1972) and wood (Gripenberg, 1952; Willför et al., 2003; Wagner et al., 2020). They have already been described in the literature as promising supplementary sources with anti-inflammatory, anticancer, antioxidant, and hepatoprotective activities (Kashyap et al., 2017; Sunil and Xu, 2019). Thus, larch industrial waste product can be used as animal supplements with no indications of adverse effects on the wellbeing of the exposed animals. It is our impression that topical applications of plant extracts and resin, as needed for the treatment of wounds, should be safe and well tolerated. We understand that mild adverse effects such as contact dermatitis are transient, can be easily detected, and can be controlled by discontinuation of a confined topical exposure.

### 3.5 *Ex Vivo* Studies

Farinacci et al. (2008) carried out an *ex vivo* analysis with sawdust extracts on ovine neutrophils, which aimed to evaluate the immunomodulatory activity by MTT assay (Table 4). 70% ethanol extract [2.23–60 μg/ml] significantly reduced the metabolism of neutrophils in a dose-dependent manner (>60%), whilst the aqueous extract [6.67–180 μg/ml] presented no effect on neutrophil viability. Activated neutrophils chemotactically migrate to the site of infection or inflammation after firm adhesion to endothelial cells followed by transmigration, production of superoxides and respiratory burst, which this study attempted to verify. The 70% ethanol extract strongly blocked neutrophil adhesion (IC_50_ 10.89 μg/ml) and inhibited the superoxide production from activated neutrophils (IC_50_ 8.15 μg/ml), concluding that the extract had anti-inflammatory activity on sheep neutrophils, possible due to the presence of flavonoids and arabinogalactan (Farinacci et al., 2008). However, these effects do not seem to be independent of cytotoxic effects and cannot be perceived as an isolated anti-inflammatory action. As described by Pferschy-Wenzig et al. (2008), the anti-inflammatory activity could be ascribed to diterpene acids, such as larixyl acetate and palustric acid, which possess anti-inflammatory activity against COX-2.

### 3.6 Antioxidant Potential

Five spectrometric analytical methods were used to evaluate the antioxidant potential of different extracts derived from *L. decidua* (Table 5). Total phenolic content (TPC) was evaluated through the Folin-Ciocalteu test, which measures the reducing power of phenolic antioxidants, mainly using gallic acid and catechins as reference standards (Munteanu and Apetrei, 2021). The antioxidant activity of plant extracts is commonly assessed by 2,2-diphenyl-1-picryl-hydrazyl-hydrate (DPPH) test, at a low cost, easy to perform and it is based on the transfer of electrons from the antioxidant source to the DPPH reagent and the result is often reported as EC_50_ (Alam et al., 2013; Munteanu and Apetrei, 2021). The ferric reducing antioxidant power (FRAP) assay evaluates the ability of antioxidants to reduce ferric iron in acid pH conditions, by an increasing of absorbance (Alam et al., 2013; Munteanu and Apetrei, 2021). The trolox equivalent antioxidant capacity measures the total antioxidant capacity to neutralize the 2,2′-azino-bis(3-ethylbenzothiazoline-6-sulfonic acid (ABTS) stable cationic radical, in which antioxidants decreases the absorption intensity (Munteanu and Apetrei, 2021). The last method was the determination of total flavonoid content (TFC) by the aluminium chloride colorimetric assay, which is the most commonly applied assay for flavonoid determination in food and plant derivatives (Pękal and Pyrzynska, 2014).

**TABLE 5:** Antioxidant evaluation of *Larix decidua* Mill. [Pinaceae] extracts.

Analytical method	Results	Authors
Total Phenolic Content (TPC)	Acetone: water 80:20 v/v extract: green cones (73.55 ± 4.11 mg GAE/g dw), mature cones (26.90 ± 5.79 mg GAE/g dw), opened cones (16.84 ± 0.90 mg GAE/g dw)	Hofmann et al. (2020)
	MeOH:water 80:20 v/v extract: Green cones (49.40 ± 0.82 mg GAE/g dw), mature cones (14.48 ± 1.95 mg GAE/g dw), opened cones (13.13 ± 0.75 mg GAE/g dw)	
	EtOH:water 80:20 v/v extract: Green cones (43.63 ± 0.38 mg GAE/g dw), mature cones (7.49 ± 0.55 mg GAE/g dw), opened cones (10.97 ± 0.09 mg GAE/g dw)	
	Bark EtOH:water 50%/50% (538 mg GAE/g dw)	Sillero et al. (2020)
	Bark: water extract (16.47% ± 0.52%); EtOH 40% (20.19% ± 1.59%); EtOH 60% (34.28% ± 0.37%); EtOH 80% (29.85% ± 0.30%) (w/w rutin)	Baldan et al. (2017)
	Bark water extract (46.7 mg epicatechin/kg dw)	Bianchi et al. (2015)
	Needle water extract (14.83 ± 0.30 mg GAE/g dw)	Dziedzinski et al. (2020)
DPPH (2,2-diphenyl-1-picrylhydrazyl) scavenging assay	Acetone:water 80:20 v/v extract: green cones (IC_50_ 13.73 ± 1.30 μg/ml), mature cones (IC_50_ 12.27 ± 1.14 μg/ml), opened cones (IC_50_ 14.39 ± 0.75 μg/ml)	Hofmann et al. (2020)
	Bark EtOH:water 50%/50% (636 mg TE/g dw)	Sillero et al. (2020)
	Bark MeOH extract (>90% GAE)	Hubert et al. (2016)
	MeOH extract: heartwood (80%), sapwood (70%), knotwood (90%), bark (90%)	Piccand et al. (2019)
	Water extract: heartwood (20%), sapwood (1%), knotwood (10%), bark (90%) (GAE)	
	Bark EtOH 40% extract (3.93 ± 0.38 μg/ml)	Baldan et al. (2017)
	Sawdust EtOH 75% v/v (9.9–15.6 μg/ml)	Hochegger et al. (2019)
	Needle water extract (326.93 ± 21.21 µM Trolox/g dw)	Dziedzinski et al. (2020)
Ferric reducing antioxidant power (FRAP)	Acetone:water 80:20 v/v extract: green cones (40.39 ± 0.73 mg AAE/g dw), opened cones (8.07 ± 0.46 mg AAE/g dw), mature cones (7.79 ± 0.52 mg AAE/g dw)	Hofmann et al. (2020)
	Bark EtOH:water 50%/50% (441 mg TE/g dw)	Sillero et al. (2020)
Total Flavonoid Content (TFC)	Bark EtOH:water 50%/50% (593 mg CE/g dw)	Sillero et al. (2020)
ABTS	Bark EtOH:water 50%/50% (1,040 mg TE/g dw)	Sillero et al. (2020)
Lipid peroxidation inhibitory assay in rat liver microsomes *in vitro*; scavenging of peroxyl radicals by chemiluminescence	Wood hexane extract followed by acetone:water (95:5 *v*/*v*) extraction showed IC_50_ value of 57 μg/L on inhibition of lipid peroxidation, 35 μg/L on scavenging of superoxide radicals, and 6.4 mmol/g on scavenging of peroxyl radicals	Willför et al. (2003)

GAE, gallic acid equivalents; TE, trolox equivalents; AAE, ascorbic acid equivalents; CE, catechin equivalents.

Comparison of results was not easy to manage, as the investigations were performed using different tree parts, different extractive solvents and ratios, and different reference standards. It is important to point out that some of the studies themselves performed comparative investigation, either by the tree part or by the extractive solvent (Table 5). The TPC is higher in green cones (73.55 ± 4.11 mg GAE/g dw) when compared to mature (26.90 ± 5.79 mg GAE/g dw) or older ones (16.84 ± 0.90 mg GAE/g dw), and also in more polar extractive solvent, such as acetone:water. After this first finding, the authors followed the DPPH and FRAP assays only with the acetone:water extracts, which demonstrated an average similar pattern for all aged cones to the DPPH assay (green cones: IC_50_ 13.73 ± 1.30; mature cones: IC_50_ 12.27 ± 1.14; and opened cones: IC_50_ 14.39 ± 0.75 μg/ml) but also a higher FRAP to the green cones (40.39 ± 0.73 mg AAE/g dw) compared to the mature (7.79 ± 0.52 mg AAE/g dw) and ripen ones (8.07 ± 0.46 mg AAE/g dw) (Hofmann et al., 2020). This publication demonstrates how important it is to investigate different extractive solvents as well as the tree parts and in different developmental stages, as an organ develops, it changes its composition and its chemical/biological outcomes. Another example is given by the DPPH assay, in which two extractive solvents (MeOH and water) were evaluated for different tree parts (heartwood, sapwood, knotwood, and bark). The MeOH extracts [heartwood (80%), sapwood (70%), knotwood (90%), bark (90%) (GAE)] were mainly more active when compared to the water extracts [heartwood (20%), sapwood (1%), knotwood (10%), bark (90%) (GAE)], and the tree parts resulted in different activities, mostly the bark as the most potent (Piccand et al., 2019).

The anti-oxidative potency of an acetone-water extract (2% v/v) was evaluated using rat liver microsomes *in vitro* (Table 5) (Willför et al., 2003). It presented IC_50_ value of 57 μg/L on lipid peroxidation, while the tested control compounds, Trolox and butylated hydroxyanisole (BHA), presented IC_50_ of 5 and 198 μg/L, respectively. The activity for scavenging of superoxide radicals was lower (IC_50_ value of 35 μg/L) than the tested control compounds (BHA and Trolox, 2.7 and 6.3 μg/L, respectively). The trapping capacity by scavenging of peroxyl radicals was 6.4 mmol/g, higher than the one of the control Trolox (8 mmol/g). Concluding the potential as a source of natural antioxidant, mainly due to the synergistic effect of phenolic compounds, such as lignans, taxifolin and secoisolariciresinol (Willför et al., 2003).

## 4 Discussion

This review resulted in a compilation of the main chemical constituents as well as the main pharmacological properties, *in vitro* and *in vivo*, described for the species *L. decidua*. As described before, European Larch resin is an oil resin, composed mainly of monoterpenes and diterpenes, among other classes of chemical compounds. Copaiba oil, an oil resin obtained from plants belonging to the genus *Copaifera*, is another example of such intricate mixtures of volatile terpenes and non-volatile terpenes (Tobouti et al., 2017; Cicek et al., 2018; Pfeifer Barbosa et al., 2019). It is therefore the aim of the following sections to present potential benefits of larch extracts for therapeutic applications. In particular, we follow up on the hypothesis that larch extracts might have a beneficial effect for the treatment of ulcerating wounds. Our key learnings can be summarized as follows:

### 4.1 Different Classes of Chemicals Contribute to the Observed Effects

Plant extracts contain a multitude of secondary metabolites. Chemical analysis detected a variety of chemical classes and provided an important piece of information for Larch (*L. decidua*). The most prevalent phytochemical class for each tree part can be summarized as follows. Bark: flavonoids, volatile terpenoids and fatty acids. Needles: flavonoids, volatile terpenoids and phenolic acids. Wood: volatile terpenoids, diterpenoids and fatty acids. Resin: diterpenoids and phenolic compounds. The class of terpenoids, especially the diterpenoids, has received most attention in studies, which have tested isolated compounds of defined chemical composition (Pferschy-Wenzig et al., 2008; Urban et al., 2016; Mulholland et al., 2017; Thuerig et al., 2018). The origins of the term terpene or terpenoid, the largest and most diverse class of plant metabolic compounds, comes from the German word turpentine—*Terpentin*—from which the first compounds of this class were isolated and structurally determined (Langenheim, 2003). The term turpentine is unspecific and is used for different types of resins, but it is known that Venice turpentine, also called larch turpentine, is derived from *L. decidua* (Scalarone et al., 2002; Dietemann et al., 2019), which has a clear and light yellowish appearance (HAB, 2014; Dietemann et al., 2019; Drugbase, 2021). Resins can be described as a lipid-soluble mixture of volatile and non-volatile terpenoid and/or phenolic compounds (Table 2), which are preformed and stored in secretory structures or may be induced at the site of an injury (Langenheim, 2003). Nevertheless, the European medicines agency (EMA, 1998) and the German Drugbase database (Drugbase, 2021) describe the composition of resin with approximately 15% of essential oils (monoterpenoids) and 50%–65% of resin acids (diterpenoids) without mentioning other potential active pharmaceutical ingredients. Thus, while terpenoids may be considered to be marker compounds for chemical standardization, they are most likely not the sole constituents contributing to the observed pharmacological actions.

### 4.2 Anti-inflammatory Effects of Resin are Often Attributed to the Action of the Diterpene Abietic Acid

Diterpenes in conifer resins are characterized to contain three main structural types, being abietanes (levopimaric acid, abietic acid, neoabietic acid, etc), pimaranes (pimaric acid, sandaracopimaric acid, isopimaric acid, etc) and labdanes (epimanool, larixol, larixyl acetate, etc) (Mills and White, 1987; Scalarone et al., 2002; Langenheim, 2003). Abietic acid is present in all parts of the tree. Recent publications have described its biological potential to be anti-inflammatory (Gao et al., 2016; Kang et al., 2018; Thummuri et al., 2018). Several studies suggest that abietic acid may interfere with signalling pathways and cytokine homeostasis. This includes inhibition of NF-κB and MAPK signalling pathways and inhibition of NFATc1 and c-Fos (Thummuri et al., 2018). This view is supported by the *in vivo* attenuation of allergic asthma in mouse, which is possibly related to the inhibition of NF-κB activation (Gao et al., 2016). Kang et al. (2018) describe activation of PPAR-γ, suppression of IL-1β, and inhibition of release of TNF-α, NO, and PGE2 by abietic acid. Therefore, abietic acid might be a promising candidate for the treatment of inflammatory disease and, as a consequence, have positive effects on wound healing. This might be cooperative effects with other larch constituents, such as larixyl acetate (see below) or taxifolin (Kolhir et al., 1996).

### 4.3 Antimicrobial Effects of Resin are Often Attributed to the Action of the Diterpene Larixyl Acetate

Larixyl acetate is one of the most described diterpenoid present in *L. decidua* in the bark (Mulholland et al., 2017; Thuerig et al., 2018), wood (Pferschy-Wenzig et al., 2008; Thuerig et al., 2018), and resin (Norin, 1972; Mills, 1973; Bol'shakova et al., 1988; Dietemann et al., 2019). Antimicrobial activity of the isolated larixyl acetate was demonstrated against *P. viticola*, with MIC_100_ of 6 μg/ml (Thuerig et al., 2018) and an efficacy of 100% at 1 mg/ml (Mulholland et al., 2017). It was therefore suggested to be effective against grapevine downy mildew, the most devastating pathogen of grapevines. It should be noted that larixyl acetate displays as well anti-inflammatory activity. These effects are mediated by inhibition of cyclooxygenase COX-2 and leukotriene LTB4 biosynthesis, with IC_50_ values of 95.1 and 10.4 µM, respectively (Pferschy-Wenzig et al., 2008). In addition, larixyl acetate and arabinogalactan, supplied as dietary supplementation in the form of larch bark for 22 days, showed modulation of cortisol concentration in sheep (Sgorlon et al., 2012). We therefore propose that the confirmed antimicrobial and proposed anti-inflammatory effects of larixyl acetate might contribute in a positive way to wound healing (Tobouti et al., 2017).

### 4.4 Larch Arabinogalactan is a Dietary Fibre With Toxin-Binding and Protective Effects on Epithelia of Endodermal Origin

Larch arabinogalactan, a FDA-approved dietary fibre, has been described in the literature to possess several biological activities, such as gastrointestinal mucosal protection, improvement of the gut microflora, stimulation of the immune system, and inhibition of metastatic tumour cells of the liver (Kelly, 1999; Kim et al., 2002; Silvani et al., 2020). Acute and prolonged toxicity tests on rats demonstrated no evidence of toxicity at a single dose of 5,000 mg/kg or with 500 mg/kg daily during 90 days, respectively (Kelly, 1999). A study of particular interest compared different natural compounds and extracts for their preventive activity on cholera or travelers’ diarrhea (Becker et al., 2010). Larch arabinogalactan and *L. decidua* sawdust showed binding to GM1-binding sites of cholera toxin. Dietary intake led to dose dependent beneficial effects (Becker et al., 2010). We therefore propose that larch arabinogalactan might have the potential to absorb bacterial toxins and to prevent bacterial invasion of wounds.

## 5 Conclusion

Our review shows that there is an increasing interest in the use of *L. decidua* and in particular in questions related to the chemical composition of its extracts. Regrettably, there was in many cases missing information, such as collection site or time of harvesting. This is a major shortcoming since this information is required to keep the traceability of the provenance of the material and to describe chemical variability due to seasonal changes and site of collection. Ethnobiological observations and approved veterinary use shows a beneficial effect of topical applications of *L. decidua* resin on wound healing. Our literature review confirms this notion and provides supportive evidence, since extracts of *L. decidua* were shown to have anti-inflammatory, anti-infective, and tissue protective effects. However, these pharmacological activities cannot be attributed to the single action of a defined chemical entity but seem to be the result of a complex interplay between different compounds. More research in the field will be necessary for an understanding of the mechanisms by which this oil resin can be used to treat ulcerating wounds. For future work we propose a differentiated pharmacological investigation of the *L. decidua*’s different components, volatile and non-volatile fractions, separately, to ascertain which chemical compounds of the extracts are responsible for specific effects and to determine if synergistic effects are playing any role. The demonstrated safety and tolerability of *L. decidua* constituents’ warrants research in this field with the prospect for the implementation of new therapeutic applications.
